# Radiogenomics for predicting p53 status, PD-L1 expression, and prognosis with machine learning in pancreatic cancer

**DOI:** 10.1038/s41416-020-0997-1

**Published:** 2020-07-21

**Authors:** Yosuke Iwatate, Isamu Hoshino, Hajime Yokota, Fumitaka Ishige, Makiko Itami, Yasukuni Mori, Satoshi Chiba, Hidehito Arimitsu, Hiroo Yanagibashi, Hiroki Nagase, Wataru Takayama

**Affiliations:** 1 Division of Hepato-Biliary-Pancreatic Surgery, Chiba Cancer Center, 666-2 Nitona-cho, Chuo-ku, Chiba, 260-8717 Japan; 2 Division of Gastroenterological Surgery, Chiba Cancer Center, 666-2 Nitona-cho, Chuo-ku, Chiba, 260-8717 Japan; 3 Department of Diagnostic Radiology and Radiation Oncology, Graduate School of Medicine, Chiba University, Inohana 1-8-1, Chuo-ku, Chiba, 260-8670 Japan; 4 Division of Clinical Pathology, Chiba Cancer Center, 666-2 Nitona-cho, Chuo-ku, Chiba, 260-8717 Japan; 5 Graduate School of Engineering, Faculty of Engineering, Chiba University, Yayoi-cho 1-33, Inage-ku, Chiba, 263-8522 Japan; 6 Laboratory of Cancer Genetics, Chiba Cancer Center Research Institute, Chiba Cancer Center, 666-2 Nitonacho, Chuo-ku, Chiba, 260-8717 Japan

**Keywords:** Cancer imaging, Cancer screening

## Abstract

**Background:**

Radiogenomics is an emerging field that integrates “Radiomics” and “Genomics”. In the current study, we aimed to predict the genetic information of pancreatic tumours in a simple, inexpensive, and non-invasive manner, using cancer imaging analysis and radiogenomics. We focused on p53 mutations, which are highly implicated in pancreatic ductal adenocarcinoma (PDAC), and PD-L1, a biomarker for immune checkpoint inhibitor-based therapies.

**Methods:**

Overall, 107 patients diagnosed with PDAC were retrospectively examined. The relationship between p53 mutations as well as PD-L1 abnormal expression and clinicopathological factors was investigated using immunohistochemistry. Imaging features (IFs) were extracted from CT scans and were used to create prediction models of p53 and PD-L1 status.

**Results:**

We found that p53 and PD-L1 are significant independent prognostic factors (*P* = 0.008, 0.013, respectively). The area under the curve for p53 and PD-L1 predictive models was 0.795 and 0.683, respectively. Radiogenomics-predicted p53 mutations were significantly associated with poor prognosis (*P* = 0.015), whereas the predicted abnormal expression of PD-L1 was not significant (*P* = 0.096).

**Conclusions:**

Radiogenomics could predict p53 mutations and in turn the prognosis of PDAC patients. Hence, prediction of genetic information using radiogenomic analysis may aid in the development of precision medicine.

## Background

Pancreatic cancer is an extremely lethal cancer, with poor prognosis and no established marker of survival. The overall 5-year survival rate is only 6%, and remains <25% even after curative surgery, thus making it one of the most lethal tumours.^1^ Recently, a whole-genome search was performed in pancreatic cancer, identifying four major genetic mutations, namely in KRAS, p53, CDKN2A and SMAD4/DPC4.^2^

In more than 90% of pancreatic cancers, mutation of KRAS has been observed. Currently, due to the high mutation rate of KRAS, it is reported that biopsy is performed to diagnose pancreatic cancer with pathological outcome and with mutated KRAS.^3^ p53, CDKN2A, and SMAD4 are tumour suppressor genes, and in pancreatic cancer, mutations have been observed in ~50–70% of p53 and in 30–50% of CDKN2A and SMAD4.^2^ In pancreatic cancer, p53 mutations are controversial, although they have been reported to correlate with worse prognosis.^4^

While the expression of programmed death ligand 1 (PD-L1) in tumour cells is considered to be a poor prognostic factor, it has attracted attention as a target and marker for anti-tumour drugs.^5^ There have been several reports of PD-L1-high expression groups being correlated with worse prognosis in pancreatic cancer.^6^ Immune checkpoint inhibitors (ICIs) that activate autoimmunity have shown effective results in lung cancer.^7,8^ They are expected to be effective in pancreatic cancer as well. Currently, there is growing expectation from precision medicine, which examines individual genetic information and uses it to analyse and select the optimal treatment for the particular patient. However, the study and availability of individualised treatments for each patient are limited by time and economy and would benefit to some extent from technical innovation in future.

Images from CT and MRI are originally qualitative data; however, they can be regarded as a matrix, since they are digital data as well. Therefore, they can be quantified using a mathematical method. Such quantitative values, namely image features (IF), can be extracted from CT and MRI data, and this research field is called radiomics. The field that integrates two different “omics” information—radiomics and genomics—is called radiogenomics.^9^ It researches for correlations between radiomics and genomics such as genomes and gene expression analysis.^9^

Radiogenomics is expected to predict the molecular profiles of tumours from image phenotypes easily, non-invasively, and inexpensively. In fact, some reports of radiogenomics have indeed predicted molecular profiles, which are clinically important in breast cancer, lung cancer, and glioblastoma, from image data.^10–12^ While there are reports predicting clinicopathological results from image data (Radiomics) in pancreatic cancer, there is no such report yet predicting genetic information like p53 and PD-L1 expression.^13–16^ The current study aimed to predict genetic information simply and inexpensively from images commonly used for cancer diagnosis and treatment. We evaluated p53 and PD-L1 expression by immunohistochemistry (IHC) and analysed their correlation with clinicopathological data, including prognosis. We examined whether the expression of p53 and PD-L1 could be predicted from CT images utilising the new field of radiogenomics.

## Methods

### Study population criteria

From January 2013 to December 2017, 140 patients were diagnosed with pancreatic cancer. Of those, 107 who did not receive preoperative chemotherapy, who followed postoperative clinical course, and who had a pathological diagnosis of pancreatic ductal adenocarcinoma (PDAC), were retrospectively examined. All patients provided written informed consent and the study was approved by our institutional ethics committee.

### Immunohistochemistry of p53 and PD-L1

We measured p53 levels by IHC using mouse monoclonal anti-human p53 protein antibody (DO7; Nichirei Biosciences Inc., Tokyo, Japan) and rabbit monoclonal anti-human PD-L1 protein antibody (SP263; Ventana Medical Systems, Inc., Tucson, AZ, USA). Five-micron-thick sections were obtained from formalin-fixed, paraffin-embedded tissues and set aside for p53 antibody (DO7) and PD-L1 (SP263) assay using a VENTANA OptiView DAB universal kit (Roche, Bazel, Switzerland) and VENTANA BenchMark ULTRA automated slide stainer (Roche, Bazel, Switzerland). Heat-induced antigen retrieval was performed using Cell Conditioning 1 (CC1; Ventana Medical Systems) for 32 min at 100 °C, followed by application of the primary antibody against p53 for 16 min at 36 °C, that of CC1 for 64 min at 100 °C, and of the primary antibody against PD-L1 for 16 min at 36 °C.

The IHC results were scored based on the percent positivity of staining. Protein expression of p53 and PD-L1 was evaluated by two pathologists as the percentage of staining area of all tumour cells. p53 status was determined by the percentage range of stained tumour cell nuclei. PD-L1 status was determined by the percentage of tumour cells with membrane staining above background.

### IHC scoring of p53, PD-L1 and variable definitions

**Fig. 1:**
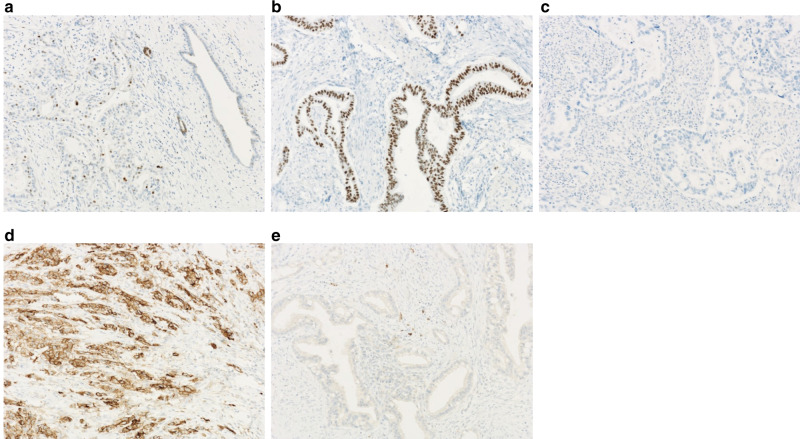
Immunohistochemistry of p53 and PD-L1 in PDAC. Typical immunohistochemical staining pattern of p53 (**a**–**c**) and PD-L1 (**d**, **e**). **a** Normal staining pattern of nuclei in tumour adjacent pancreatic tissue and “negative” staining pattern in PDAC for p53 in IHC. **b** Abnormal staining pattern in PDAC; nuclear accumulation of p53 protein was observed in IHC, which was defined as “Positive” indicating mutated p53. C, Absence of p53 in PDAC, which was also defined as “Positive”. Example of typical immunohistochemical “Positive” and “Negative” staining pattern of PD-L1, respectively (**d**, **e**). Image magnification of ×400.

In normal pancreatic tissues adjacent to the tumour, nuclear accumulation of p53 was observed in pancreatic ductal cells with scattered, non-specific weak nuclear staining using IHC (Fig. 1a); this was considered the negative control.^4,17^ p53 mutations resulted in either nuclear accumulation of p53 protein, which was defined as the overexpression type (Fig. 1b), or in the complete absence of p53, defined as the null type (Fig. 1c), in contrast to the negative control.^4,17^ Wild-type p53 was characterised by a staining pattern in tumour nuclei that was equivalent to the negative control (Fig. 1a). For statistical comparisons, cases with p53-stained nuclei of total tumour cells exceeding 20% or completely absent in tumour cells were defined as “p53-positive”. There was no uniform opinion about how to evaluate PD-L1 expression in IHC.^6^ According to previous reports, the threshold for staining percentage of tumour cells was set at 1–10%, and cases with PD-L1-stained cells >1% of the total tumour cells were considered as PD-L1-positive (Fig. 1d, e).^6^ An 80% agreement among the pathologists involved in immunostaining evaluation was set as the criterion. When pathologists disagreed with regard to an evaluation, a decision was reached based on consultation.

### CT acquisition

All CTs were performed using a 128-detector-row CT system (SOMATOM Definition Flash; Siemens; Erlangen, Germany). The following imaging parameters were applied: tube voltage, 120 kVp; tube current, 160 mAs; beam pitch, 0.6; and resolution 0.68 × 0.68 × 5 mm. Contrast agent (Iopamidol, Iopamiron 300; Bayer, Leverkusen, Germany; 100 mL) was administered through the superficial vein of upper extremity using double-head power injector (body weight ≥ 55 kg; 150 ml injected at 4.5 mL/s, body weight < 55 kg; 100 ml injected at 3.6 ml/s). The application of contrast agent was followed by that of normal saline (30 mL) at the same injection rate. After injection of the contrast agent, two-phase images of imaging slices taken at 40 and 120 s were used for analysis.

### Tumour segmentation

**Fig. 2:**
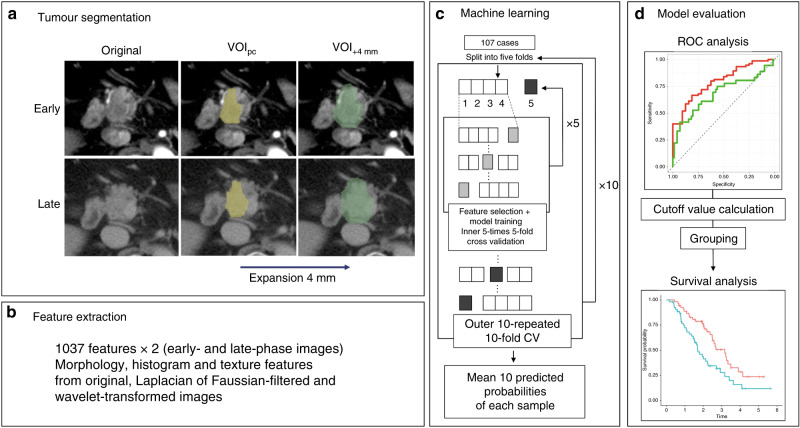
Machine learning processing was summarised. Total 2,074 IFs extracted from two- phase CT images. Predictive models for p53 and PD-L1 were constructed with machine learning from the IFs. The results were visualised and interpreted in AUC plots and Kaplan–Meier plots.

A board-certified diagnostic radiologist and surgeon (15 and 7 years of experience in pancreatic imaging, respectively) delineated the volume of interest in pancreatic cancer (VOI_pc_) in early- and late-phase images individually (Fig. 2).

If available, magnetic resonance imaging and ^18^F-positron emission tomography were referenced. Next, VOI_+4mm_ was created by mechanically expanding the axial plane only by 4 mm around each VOI_pc_. VOI_+4mm_ included the tumour and peritumoural region seen in CT.

### Imaging feature extraction

Imaging features were extracted using an open-source python package, PyRadiomics v2.2.0 (http://www.radiomics.io/pyradiomics.html).^18^ Pyradiomics can calculate various quantitative values from images using various mathematical methods based on morphological, histogram and texture analyses. The quantitative values reflect the imaging characteristics of the tumour, such as heterogeneity. Absolute rescaling method (−150 to 500 Hounsfield unit) was applied. Pixel values between the upper and lower limits were resampled into 64 levels and those outside the limits were truncated. Morphology, histogram, and texture features were calculated from original images. The same types of features were extracted from the Laplacian of Gaussian-filtered and wavelet-transformed images. Finally, a total of 1037 features were extracted from each VOI (Fig. 2).

### Variable definitions

For statistical survival analysis, age, preoperative carcinoembryonic antigen (CEA), and preoperative CA19-9 were divided into two groups with 70, 3.3, and 137.4 as the median, respectively. For surgery time and blood loss, we compared the survival rates in both groups (311 min, 600 ml). We performed pancreaticoduodenectomy (PD), distal pancreatectomy (DP), and total pancreatectomy (TP) for PDAC. In addition, we adopted a unified procedure at the Department of Hepatobiliary and Pancreatic Surgery in our hospital. Lymph nodes, margin status, cytology, lymphatic invasion, neural invasion, vascular invasion, differentiation, and TNM staging (UICC 8th edition) were defined based on the pathological results. Lymph nodes were either positive or negative for lymph node metastasis. Vascular invasion was divided into two groups: v0, v1 and v2, v3, since there was only one patient in v0. Differentiation was divided into two groups for survival analysis, namely “well” and “moderate/poor”.

### Statistics

Significance of the difference between the status of p53, PD-L1 (positive/negative), and several clinical and pathologic variables was assessed by the *χ*^2^ test, Fisher’s exact test, or Mann–Whitney *U* test. Overall survival (OS) was defined as the period between surgery and final observation (in days). A survival curve was prepared using the Kaplan–Meier method, and log-rank test assessed the significant differences. Multivariate analysis was performed using the Cox regression model to study the significant factors in log-rank test. A *P*-value < 0.050 was considered significant.

### Machine learning

Feature selection consisted of two steps to stabilise the predictive power of the model. First, Mann–Whitney *U* test was performed on each imaging feature, and only those with significant difference were retained. Second, another feature selection with recursive feature elimination was performed using random forest function. Finally, 2074 features derived from early and late phases were put into XGBoost to construct the predictive models for p53 and PD-L1, respectively. The feature selection and model construction steps were performed with nested cross validation. Inner cross validation for feature selection was 5-repeat 5-fold and outer cross validation for model construction was 10-repeat 5-fold (Fig. 2).

### Model evaluation

The mean output values of 10 repeats were used for receiver operating characteristic (ROC) analysis. To evaluate the survival prediction of machine learning models, cut-off values were defined from the point closest to the top-left part of ROC plot with perfect sensitivity and specificity. Log-rank test was performed between two groups, defined by predicted p53 and PD-L1, respectively. All statistical analyses and machine-learning were conducted using R version 3.5.1 (R Foundation for Statistical Computing, Vienna, Austria). The summary of the processing is shown in Fig. 2.

## Results

### Patient background

From January 2013 to March 2018, 140 patients were diagnosed with pancreatic cancer, after surgery, based on pathological diagnosis. Of those, 22 patients were excluded, since they had received preoperative chemotherapy or chemoradiation. Four cases were excluded owing to atypical pancreatic cancer. Three cases were diagnosed as having intra-ductal papillary mucinous carcinoma with infiltration components; since the infiltration site was slight, the remaining samples could not be evaluated. One patient was excluded owing to liver metastasis at the time of surgery. The following cases were also excluded: one patient with oesophageal cancer, one with gastric cancer, and one with pancreatic recurrence referred to the current hospital from another. A retrospective study was conducted on 107 out of 140 subjects. The observation period was from January 2013 to July 2019, with a median of 708 days (58–2067 days). The median age was 70 years (50–87 years), and gender ratio was 60:47. There were 70 cases with PD (Pancreaticoduodenectomy), 35 cases with DP (Distal Pancreatectomy), and two cases with TP (Total Pancreatectomy). The median operation time was 311 min (121–586 min) and median blood loss was 600 ml (35–4600 ml). The median values for preoperative CEA and CA19-9 were 3.3 (0.5–47.3) and 138.4 (0–47588.2), respectively. Ninety-three patients were negative for intraoperative peritoneal washing cytology. The curative resection was R0 in 89 cases, and the histological types were well, moderate, and poor in 46, 53, and 8 cases, respectively. Lymphatic infiltration (ly0) and nerve infiltration (ne0) were negative in 29 and 6 cases, respectively. Venous invasion was negative in only 1 case, and v1 were 20 cases. Lymph node metastasis was found in 76 cases. In T factor (UICC 8th edition), T2 was the maximum in 60 cases. In TNM classification (UICC 8th edition), Stage III was the most common in 39 cases, followed by Stage III in 37 cases (Table 1).

**Table 1:** Clinocopathological Parameters and p53 status, PD-L1 status.

	p53 status		*P* value	PD-L1 stasus		*P* value
	Negative *N*(%)	Positive *N*(%)		Negative *N*(%)	Positive *N*(%)	
Sex						
Male	23 (21.5)	37 (34.6)		35 (32.7)	25 (23.4)	
Female	9 (8.4)	38 (35.5)	0.036	36 (33.6)	11 (10.3)	0.064
Age						
70 (50–87)	69 (50-83)	71 (51-87)	0.319	69 (51-87)	72 (50-82)	0.087
Preoperative CEA						
3.3 (0.5–47.3)	2.95 (0.5–28.5)	3.4 (0.7–47.3)	0.448	3 (0.5–28.5)	3.7 (0.8–47.3)	0.102
Preoperative CA19-9						
138.4 (0–47588.2)	115.9 (0–32951.5)	139 (0–47588.2)	0.718	85.4 (0–47588.2)	236.8 (2–31800.7)	0.060
Operation type						
PD	20 (18.7)	50 (46.7)		47 (43.9)	23 (21.5)	
DP	12 (11.2)	23 (21.5)		23 (21.5)	12 (11.2)	
TP	0 (0)	2 (1.9)	0.755	1 (1.0)	1 (1.0)	1.000
Cytology						
Negative	28 (26.2)	65 (60.8)		61 (57.0)	32 (29.9)	
Positive	4 (3.7)	10 (9.4)	1.000	10 (9.3)	4 (3.7)	0.769
Margin status						
R0	27 (25.2)	62 (57.9)		61 (57.0)	28 (26.3)	
R1	4 (3.7)	12 (11.2)		10 (9.3)	6 (5.6)	
R2	1 (1.0)	1 (1.0)	0.689	0 (0)	2 (1.9)	0.159
Differenciation						
Well	15 (14.0)	31 (29.0)		34 (31.8)	12 (11.2)	
Moderate	15 (14.0)	38 (35.5)		33 (30.8)	20 (18.7)	
Poor	2 (1.9)	6 (5.6)	0.898	4 (3.7)	4 (3.7)	0.272
Lympathic invasion						
Negative	8 (7.5)	21 (19.6)		21 (19.6)	8 (7.5)	
Positive	24 (22.4)	54 (50.5)	0.816	50 (46.7)	28 (26.2)	0.495
Vascular invasion						
Negative(0/1)	8 (7.5)	13 (12.2)		15 (14.0)	6 (5.6)	
Positive	24 (22.4)	62 (57.9)	0.427	56 (52.3)	30 (28.0)	0.797
Neural invasion						
Negative	2 (1.9)	4 (3.7)		5 (4.7)	1 (1.0)	
Positive	30 (28.0)	71 (66,4)	1.000	66 (61.7)	35 (32.7)	0.661
Lymph node metastasis						
Negative	8 (7.5)	23 (21.5)		27 (25.2)	4 (3.7)	
Positive	24 (22.4)	52 (48.6)	0.645	44 (41.1)	32 (29.9)	<0.001
pT(UICC) 8th						
T1	6 (5.6)	13 (12.2)		15 (14.0)	4 (3.7)	
T2	20 (18.7)	40 (37.8)		40 (37.4)	20 (18.7)	
T3	6 (5.6)	22 (20.6)	0.545	16 (15.0)	12 (11.2)	0.320
pStage(UICC 8th)						
IA	4 (3.7)	8 (7.5)		12 (11.2)	0 (0)	
IB	3 (2.8)	11 (10.3)		11 (10.3)	3 (2.8)	
IIA	1 (1.0)	4 (3.7)		4 (3.7)	1 (1.0)	
IIB	12 (11.2)	25 (23.4)		24 (22.4)	13 (12.2)	
III	12 (11.2)	27 (25.2)	0.946	20 (18.7)	19 (17.8)	0.013

*UICC* Union for International Cancer Control.

### p53 immunostaining

Seventy-five cases (70.0%) were p53 positive. There was a difference in gender ratio between p53 positive and negative (*P* = 0.036); however, there was no difference in other clinicopathological factors such as preoperative tumour markers and lymph node metastasis (Table 1).

### PD-L1 immunostaining

Thirty-six cases (33.6%) were PD-L1 positive. Lymph node metastasis was more frequent in the PD-L1-positive group (*P* < 0.001), and a tendency for higher stage cases in PD-L1-positive group was observed in TNM staging (UICC 8th edition). There was no difference between other factors, including histological types, in PD-L1 (Table 1).

### Relationship between clinicopathological factors and prognosis

We examined the relationship between clinicopathological factors and prognosis using the log-rank test. There was no difference in survival rates based on gender or age (Table 2). CEA and CA19-9, known as preoperative tumour markers, were analysed and the high-CA19-9 group was significantly related to poor prognosis in PDAC (*P* = 0.004). In the surgical procedure, DP/TP and long-term operation significantly worsened prognosis (*P* = 0.001, 0.001). Intraoperative peritoneal washing cytology and margin status histology showed no clear deterioration in prognosis, although the latter significantly worsened in venous invasion and nerve invasion groups (*P* = 0.013, 0.043). Moreover, prognosis was significantly worse in the lymph node metastasis-positive group and T3 group (UICC 8th edition) (*P* < 0.010, 0.018), and similar tendency was observed in TNM staging (Table 2).

**Table 2:** Univariate analysis of prognostic factors for OS.

Variable	No. of patients (%)	Univariate analysis for OS	
		Median (95% confidence interval) (days)	Log-rank (*P* value)
Gender			
Male	60 (56.1)	823 (622–1065)	0.407
Female	47 (43.9)	990 (496–1324)	
Age			
≤70	55 (51.4)	911 (711–1218)	0.402
>70	52 (48.6)	823 (599–1065)	
Range	50–87		
Follow-up (days)			
Median	708		
Range	58–2067		
Preoperative CEA			
≤3.3	54 (50.0)	1155 (730–1276)	0.067
>3.3	53 (50.0)	746 (572–905)	
Preoperative CA-19-9			
≤137.4	54 (50.0)	1156 (711–1218)	0.004
>137.4	53 (50.0)	711 (599–1065)	
Operation type			
PD	70 (65.4)	711 (534–905)	0.001
DP/TP	37 (34.6)	1324 (963-NA)	
Operation time			
≤311	55 (51.4)	1276 (864–1495)	0.001
>311	52 (48.6)	656 (515–905)	
Bleeding volume			
≤600	54 (50.5)	1065 (730–1495)	0.087
>600	53 (49.5)	777 (560–942)	
Cytology			
CY0	91 (88.3)	911 (302–500)	0.069
CY1	12 (11.7)	454 (106–484)	
Margin status			
R0	86 (83.5)	905 (268–498)	0.812
R1/R2	17 (16.5)	942 (135–643)	
Differentiation			
Well	47 (43.9)	1156 (711–1218)	0.087
Moderate/poor	60 (56.1)	24.9 (599–1065)	
Lymphatic invasion			
Negative	30 (28.0)	1175 (572-NA)	0.148
Positive	77 (72.0)	823 (622–979)	
Neural invasion			
Negative	6 (6.5)	NA (1175-NA)	0.013
Positive	101 (93.5)	813 (656–979)	
Vascular invasion			
Negative (v0/1)	21 (19.6)	1175 (560-NA)	0.043
Positive (v2/3)	85 (80.4)	823 (547–905)	
Lymph nodes			
Negative	31 (29.0)	1424 (990-NA)	<0.001
Positive	76 (71.0)	735 (547–905)	
T factor (UICC 8th)			
T1/2	79 (76.7)	963 (777–1218)	0.018
T3	28 (26.2)	572 (304–979)	
Stage (UICC 8th)			
IA	12 (11.2)		0.055
IB	14 (13.1)		(I,IIvs III)
IIA	5 (4.7)		
IIB	37 (34.6)	942 (615–1175 (I,II))	
III	39 (36.4)	572 (362–823 (III))	
p53 IHC			
Negative	32 (46.6)	1218 (979–1512)	0.008
Positive	75 (53.4)	735 (560–911)	
PD-L1 IHC			
Negative	71 (66.4)	1065 (800–1218)	0.013
Positive	36 (33.6)	560 (362–823)	

For statistical analysis, each stage (UICC 8th) was divided into two groups: I, II and III.

*OS* overall survival, *NA* not available, *UICC* Union for International Cancer Control, *IHC* immunohistochemistry, *T1/2* T1 and T2.

### Relationship between p53 and PD-L1 expression (by immunostaining) and prognosis

**Fig. 3:**
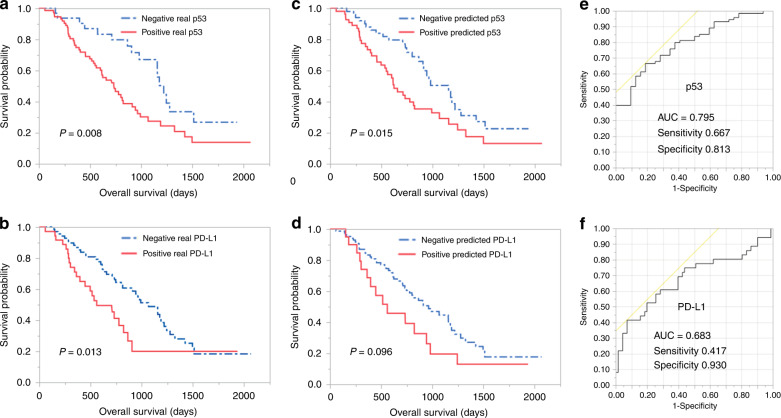
Kaplan-Meier plots of p53 and PD-L1 status by IHC compared with status by machine-learning, and ROC curve. Kaplan–Meier plots for patients with PDAC demonstrating prognostic influence for the “real” status (positive/negative) of p53 in IHC (**a**), PD-L1 in IHC (**b**) and “predicted” status of p53 (**c**), PD-L1 (**d**) with reference to overall survival. ROC curve was constructed by “real” status and “predicted” status of p53 (**e**) and PD-L1 (**f**), respectively. “predicted” status was calculated with machine learning and 1037 IFs extracted from CT.

We investigated the correlation between clinicopathological factors, including p53 and PD-L1 expression, and prognosis. The p53-positive and PD-L1-positive groups were associated with poor prognosis (*P* = 0.008, 0.013) (Fig. 3a, b).

### Examination of prognostic factors by multivariate analysis

In the 107 patients studied, we found p53, PD-L1, CA19-9, surgical time, operative procedure, venous invasion-positive, nerve invasion-positive, lymph node metastasis, and T factors to be significantly associated with poor prognosis; of these nine factors, six with the lowest P values (p53, PD-L1, CA19-9, operation time, operative procedure, and lymph node metastasis) underwent multivariate analysis. All factors except the operation time were found to be significantly associated with poor prognosis (Table 3).

**Table 3:** Multivariate analysis of prognostic factors for OS.

Multivariate analysis of prognositc factors for OS			
	OS		
Variables	Hazard ratio	95% confidence limit	*P* value
Preoperative CA-19-9			
≤137.4 (*n* = 54)	1		
>137.4 (*n* = 53)	2.157	1.293–3.597	0.003
Operation type			
PD (*n* = 70)	1		
DP/TP (*n* = 37)	0.4063	0.205–0.805	0.010
Operation time			
≤311 (*n* = 55)	1		
>311 (*n* = 52)	1.376	0.771–2.456	0.280
Lymph node			
Negative (*n* = 31)	1		
Positive (*n* = 76)	4.058	1.969–8.362	<0.001
p53 status			
Negative (*n* = 32)	1		
Positive (*n* = 75)	2.802	1.532–5.127	0.001
PD-L1 status			
Negative (*n* = 71)	1		
Positive (*n* = 36)	1.970	1.094–3.547	0.024

*OS* overall survival, *PD* pancreatoduodenectomy, *DP/TP* distal pancreatectomy and total pancreatectomy.

### Predictive power of machine learning models

Area under the curve (AUC) for p53 was 0.705 and 0.795 with imaging features of VOI_pc_ and VOI_+4mm_, respectively (Fig. 3e). The AUC for PD-L1 was 0.660 and 0.683 with imaging features of VOI_pc_ and VOI_+4mm_, respectively (Fig. 3f). Overall survival was significantly different between the groups defined by the predicted status of p53 (positive/negative) using machine learning (*P* = 0.015) (Fig. 2c). In contrast, the predicted status of PD-L1 divided the groups into better and worse prognosis, although the difference was not significant (*P* = 0.096) (Fig. 3d).

## Discussion

We confirmed the positive expression of abnormal p53 and PD-L1 in IHC to be an independent prognostic factor in PDAC. Furthermore, we examined whether the expression of p53 and PD-L1 could be predicted using radiogenomic analysis. Results clearly revealed the expression of p53 to be predictable with certain accuracy.

Although p53 regulates the cell cycle, DNA-damaged cells can lead to apoptosis. When p53 is mutated, it abnormally proliferates and participates in carcinogenesis. Approximately 70–90% of p53 mutations are found in PDAC, and it is believed that if a therapeutic drug for p53 was available, it would have a great effect on PDAC.^2,19^ Recently, there have been reports of agents that restore p53 function, showing effectiveness in oesophageal squamous cell carcinoma, osteosarcoma, multiple myeloma, lung cancer, breast cancer, and colon cancer in vitro.^20–24^

In this study, we used immunohistochemistry to determine the presence of p53 mutations. In patients with PDAC, the rate of abnormal expression of p53, as seen by immunohistochemistry, was reported to be 50–90%, whereas Ohshima et al. had reported it to be 81.8%.^2–4,25^ In this study, positive p53 was seen in 75 cases (70%), almost in agreement with the previous report.^4,26^ Lack of p53 protein (null type) is associated with frameshift and nonsense mutations, whereas p53 over expression occurs (overexpression type) as a result of missense mutations.^27,28^ Further, in the case of pancreatic cancer, Schlitter et al. showed a relationship between p53 mutations and a p53 positive status on IHC (null type and overexpression type).^26^ In their study, p53 mutations were found overall in 77.6% of the PDAC cases. Of these, 26.6% were null type, which were associated with intragenic deletions, nonsense mutations, frameshifts, or splice site mutations in p53, while 51% were overexpression type, which were associated with missense mutations.^26^ The results indicated that a p53 positive status on IHC reflects the presence of p53 gene mutations.^26^ We found similar frequencies of p53-positive PDAC cases in our study—null and overexpression types in 20 (18.7%) and 55 (51.3%) cases, respectively. Other studies have also shown that p53 positive status on IHC correlates with p53 mutations. In fact, for colon, breast, ovarian, and bladder cancer, p53 mutations have been defined by p53 positive status on IHC, and this information has been used to predict prognosis in these cancers.^4,28–33^

There is no consensus yet for demonstrating and quantifying PD-L1 expression in PDAC, although the expression rate of PD-L1 in tumour cells is regarded as ~4–60%; however, there is no clear threshold to define PD-L1 positivity.^10,34^ In this study, we defined PD-L1-positive cases as the ones in which IHC-stained cells were 1% or more of all tumour cells; the group of PD-L1-positive cases is correlated with vascular invasion, histological type, and lymph node metastasis, although the reports have not been consistent. Results of the current study correlated with lymph node metastasis.^1,6^ PD-L1 is a marker for ICIs that have shown significant results in lung cancer.^5,7,8^ In recent years, ICI has been suggested to have an effect on PDAC, and hence, has attracted remarkable attention. However, clinical trials have shown the clinical effect of anti-PD-L1 antibody alone to be of little benefit, although the number of cases reported was small.^35^ Recent reports have indicated ICI to be effective in colorectal cancer with high frequency microsatellite instability (MSI-H), known to be present in ~1.5% of all colorectal cancers.^36^ The instability may be expected to occur in ~2% of PDACs as well, and the effect of ICI may also be expected similarly. It may be effective when administered to patients with high PD-L1 expression or when combined with other molecular targeting drugs, chemotherapy, or radiation therapy.

As described above, it is obvious that p53 and PD-L1 may be important factors in the diagnosis and treatment of PDAC at present, as well as in the future. However, examining the expression and mutation of these factors using molecular biology techniques would require more time and expenses, owing to which an alternative imaging approach would be preferable. If this can be made feasible, it will have immense clinical significance. Radiogenomics is being increasingly reported in malignant tumours other than PDAC. In breast cancer, Genevieve et al. had predicted the Oncotype DX Test Recurrence Score from IFs extracted from mammography and MRI, and showed it to be a convenient biomarker for recurrence.^10^ Hectors et al. had reported the analysis of digital data in MRI to possibly predict the expression of vascular endothelial growth factor A (VEGFA) and immune checkpoints, differentiation cluster 274 (CD274), and cytotoxic T lymphocyte-associated protein 4 (CTLA4) in hepatocellular carcinoma.^37^ Taguchi et al. had reported KRAS mutations in colorectal cancer predicted from CT images using AI technology.^38^ A previous report had identified blood microRNAs associated with prognosis in oesophageal squamous cell carcinoma and predicted microRNA level and prognosis from IFs based on CT.^39^ Although there was only one report on pancreatic cancer, Attiyeh et al. reported that in 35 cases, only 28 IFs were significant for SMAD4, out of 255 IFs extracted from CT. Using a two-dimensional scale, difference in the distribution of SMAD4 between normal and abnormal cases could be demonstrated, and the status of SMAD4 (normal / abnormal) could be identified by CT; the proportion of stroma could also be predicted from IFs.^15^ However, the number of cases was 35, and the number of IFs used for analysis was strictly limited to 28. Although radiogenomic analysis had been performed by various approaches, there has been no report yet on radiogenomic analysis predicting genetic information from IFs using machine learning in pancreatic cancer.

In this study, it was possible to predict p53 status (positive/negative) with some accuracy using IFs extracted from CT images and radiomics analysis. However, AUC was not sufficient with VOI_pc_ set at the tumour margin of PDAC, and a sufficient AUC could be obtained with VOI_+4mm_ set in the region including the tumour periphery area and additional 4 mm. This implied that VOI_pc_, which captured only the outline of tumour in the CT image, might reflect only the solid content of PDAC, and might not include all the tumour with the peripheral invasive part. Hutchings et al. had suggested p53 mutation to be associated with carcinogenesis, spread, and progression to the pancreatic duct surrounding PDAC.^40^ Moreover, other reports had suggested p53 mutation to promote tumour cell invasion and metastasis via cancer-associated fibroblasts.^27^ These results indicated p53 mutation to possibly affect the major margins. Therefore, representing such properties of p53 and including the surroundings around the outline of tumour image might have caused the enhanced detection sensitivity in radiomics analysis.

The PD-L1 status (positive/negative) could not be adequately predicted by image properties (AUC = 0.660); predictions including analysis around the tumour (+4 mm) were also not sufficient (AUC = 0.683). An earlier report had suggested PD-L1 to affect histology, lymph node metastasis, and vascular invasion; however, it was inconsistent and reported PD-L1 to affect the tumour microenvironment.^1,6^ On the other hand, there was no report regarding the effects on extratumoural progression.^34,35,41,42^ The features of PD-L1-positive/-negative cases may not be well reflected in CT images. While p53 is a molecule that exists upstream in many genetic cascades (also called “genomic guardian”) and has multiple functions in cancer suppression, PD-L1 is a downstream molecule with the distinct function of immunosuppression.^43^ This difference may introduce a discrepancy in their influence on the image.

This study had some limitations, first being the small number of cases (107 cases). Second, this was a retrospective study performed at a single centre. Larger prospective studies in collaboration with other centres should be conducted to further confirm the results of our study. In addition, p53 gene mutations were evaluated only indirectly using IHC. In the future, the frequency and type of p53 mutations present in PDACs could be directly assessed using next-generation sequencing.

Here, we used only CT images for PDAC using radiomics analysis, whereas PDAC may have many indistinguishable lesions and may require image information from another modality with high contrast resolution such as MRI. Although VOIs were created by skilled radiologists and surgeons, and PDAC had many unclearly defined lesions, VOIs might have been chosen with bias, which could be an issue in future considerations.

## Conclusion

In this study, occurrence of p53 gene mutation and abnormal PD-L1 expression was examined using IHC. Positive p53 and PD-L1 were independent factors that worsened prognosis. Radiogenomic analysis using CT images was able to predict the presence of p53 mutations, and hence, disease prognosis. Prediction of genetic information from these images using radiogenomics may help both precision and personalised medicine.

## Data Availability

The data that support the findings of this study are available from the corresponding author upon reasonable request.
